# Supplementary material for: Early-life colonization by enterotoxigenic Bacteroides fragilis remodels gut epithelial stem cell states to drive colorectal cancer susceptibility
Source: bioRxiv. 2026 Sep 29:2026.09.28.755180. Preprint. [Version 1] doi: 10.64898/2026.09.28.755180 (PMC13648629; doi:10.64898/2026.09.28.755180)
Supplement: Supplementary file 1 [file media-1.pdf]

Supplementary Information

Early-life bacterial toxin exposure remodels gut epithelial stem cell fate to drive cancer susceptibility

Seongmi K. Russell[1], Ezequiel Valguarnera[1], Jesse J. Pak[1], Juliane Bubeck Wardenburg[1]*

Corresponding author: Email: jbubeck@wustl.edu

The PDF file includes:

Supplementary Figs. 1-3

Supplementary Tables 1-3

Other Supplementary Materials for this manuscript include the following:

Supplementary Movies 1-2

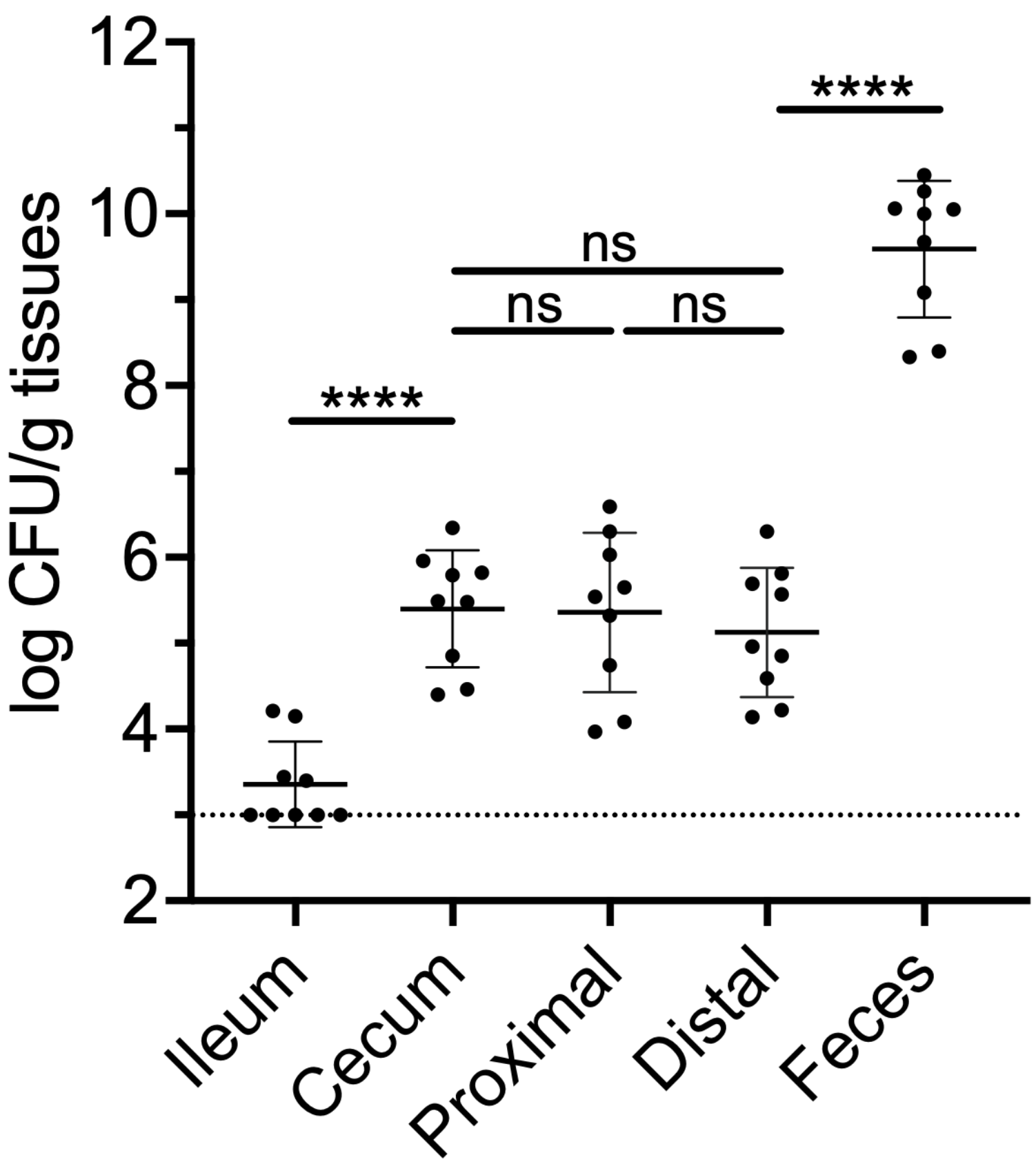

Supplementary Fig. 1. Quantification of ETBF tissue colonization across intestinal regions, compared with fecal titers. Intestinal tissues were extensively washed with PBS (10–15 times per cycle, 3 cycles total) to remove luminal bacteria, including those within the mucus layer, prior to homogenization. Tissue homogenates were serially diluted and plated for colony-forming unit (CFU) enumeration. Data represent CFU per tissue from the indicated intestinal locations. ****$p < 0.0001$, ns = not significant.

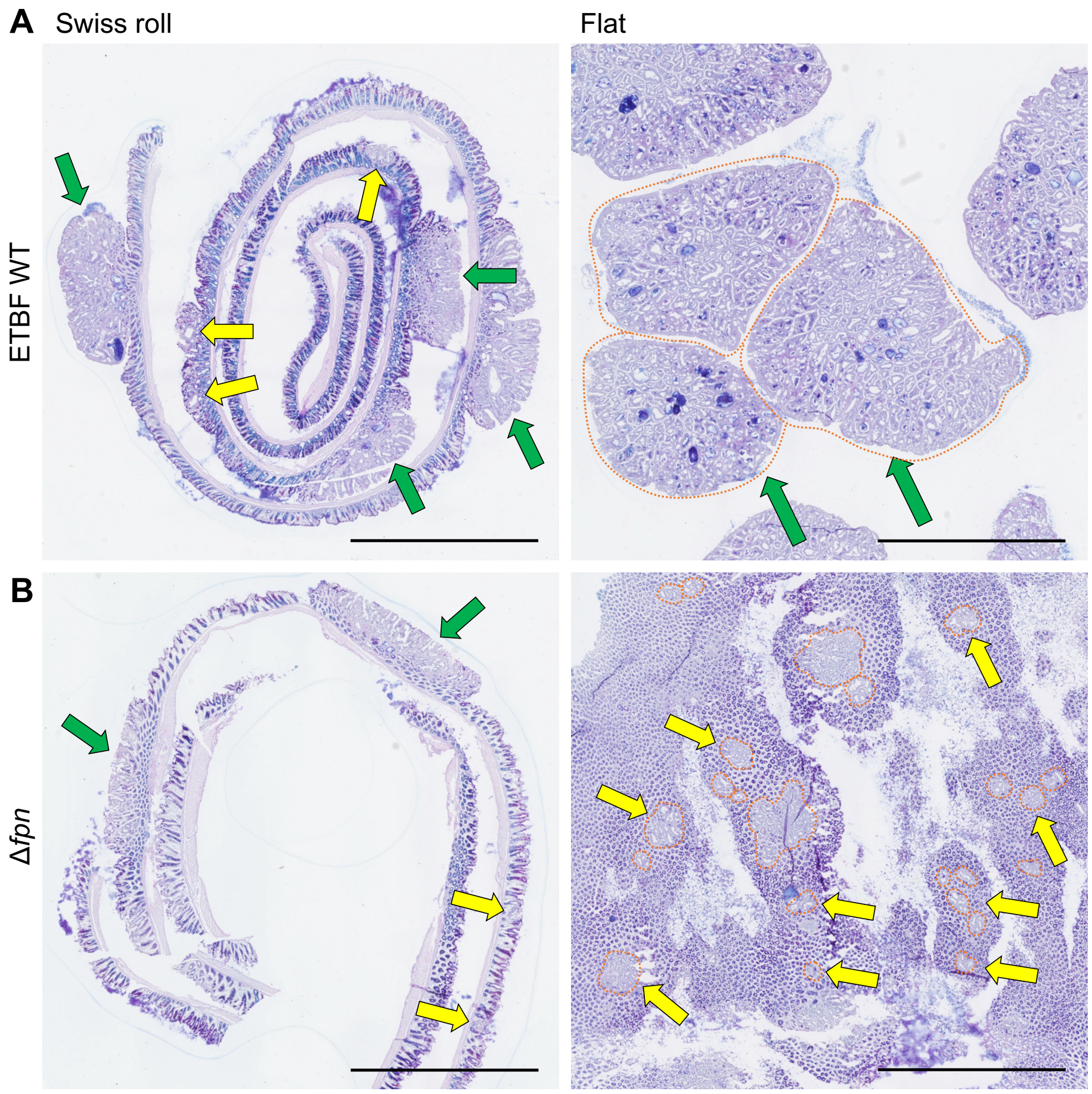

Supplementary Fig. 2. Visualization of precancerous lesions in distal colon tissue. Distal colons from 8-week-old *Apc*$^{\mathrm{Min/+}}$ mice neonatally colonized with ETBF WT or Δ*fpn* strains were analyzed histologically and by confocal microscopy. (A-B) AB/PAS-stained Swiss-roll (left) and flat-mounted (right) sections show aberrant crypt foci (ACF; yellow arrows) and early polyps (green arrows) in colons colonized with (A) ETBF WT or (B) Δ*fpn*.

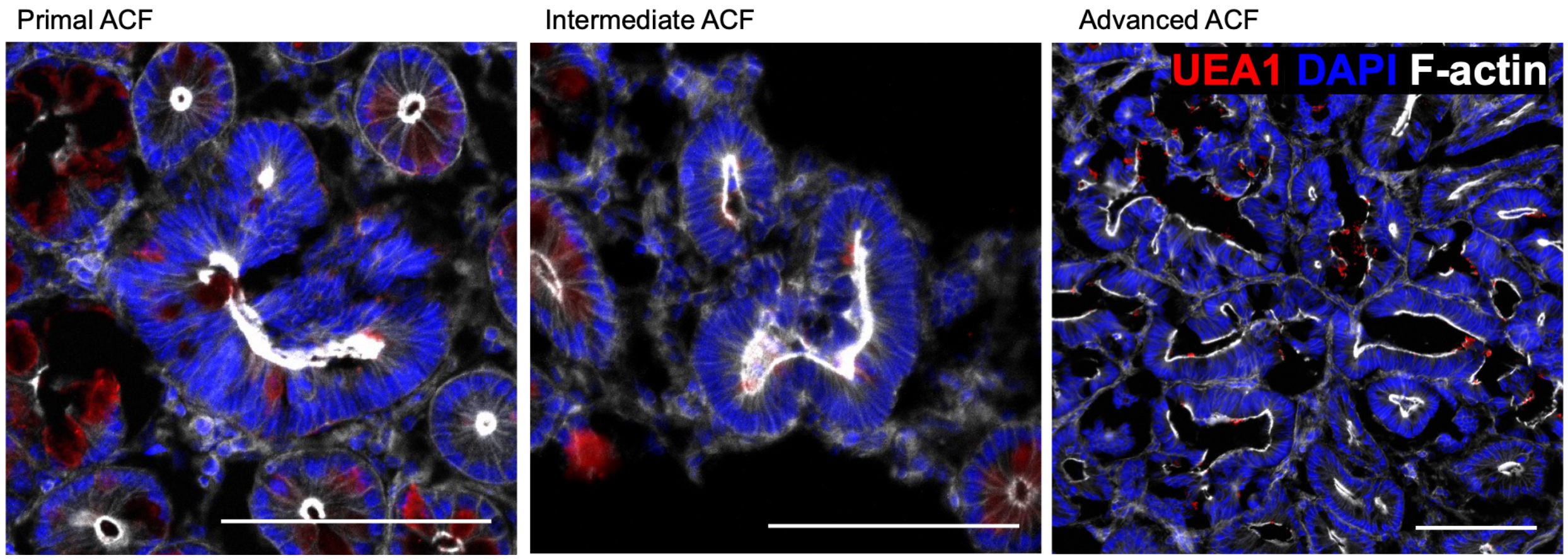

Supplementary Fig. 3. UEA1 staining confirms reduced goblet cell-associated mucin signal in ETBF-induced ACF lesions, related to Fig. 3. Representative confocal images of flat-mounted distal colon tissue from ETBF-colonized *Apc*$^{Min/+}$ mice stained with UEA1 (red), DAPI (blue), and F-actin (white), showing primal, intermediate, and advanced ACF lesions. ACF lesions show reduced UEA1 signal compared to adjacent normal crypts. Scale bars, 100 µm.

Supplementary Table 1.

Bacterial strains used in this study.

Strain	Sources	Identifier	Variants
B. fragilis (ETBF)	ATCC;<br>This study;<br>Hill et al., 2024 (*30*);<br>Hecht et al., 2016 (*31*)	ATCC 43859	GFP $Tet^R Clin^R$<br>Δ*bft* GFP $Tet^R Clin^R$<br>Δ*fpn* GFP $Tet^R Clin^R$
B. fragilis (NTBF)	ATCC;<br>Hill et al., 2024 (*30*);<br>Hecht et al., 2016 (*31*)	TM4000/638R	RFP $Rif^R Clin^R$<br>Δ*tssC* RFP $Rif^R Clin^R$

Supplementary Table 2.

Mouse strains and genotypes used in this study. R26-LSL-H2B-mCherry mice were crossed with Atoh1-Cre mice to generate Atoh1 (R26-LSL-H2B-mCherry; Atoh1-Cre) reporter mice used in this study.

Mouse line	Description / Genotype	Experimental use	Source / Reference
C57Bl/6J	Inbred wild-type background strain	Baseline control and background strain; used for breeding and neonatal colonization studies	The Jackson Laboratory (000664)
Lgr5-EGFP-IRES-CreERT2	Knock-in Lgr5-EGFP reporter	Lineage tracing and quantification of stem-cell expansion	JAX (008875)
TCF/Lef:H2B-GFP	Nuclear GFP reporter of Wnt co-factor TCF/Lef	Detection of Wnt activation	JAX (013752)
Atoh1-Cre	Cre recombinase knocked-in at the *Atoh1* locus	Secretory-lineage-specific recombination for epithelial tracing	JAX (011104)
R26-LSL-H2B-mCherry	Rosa26-loxP-STOP-loxP nuclear mCherry reporter	Visualization of Cre-dependent labeling of epithelial lineages	JAX (023139)

Apc$^{Min/+}$	Germline truncating mutation in *Apc* tumor-suppressor gene	Model of spontaneous intestinal adenoma and ETBF-driven tumorigenesis	JAX (002020)

Supplementary Table 3.

Antibodies and probes used for immunofluorescence.

Target	Antibody / probe	Dilution	Source
F-actin	Phalloidin (Alexa Fluor™ 555)	1:250	Invitrogen A34055
F-actin	Phalloidin (Alexa Fluor™ 647)	1:250	Invitrogen A22287
Nucleus	ProLong™ Gold Antifade Mountant with NucBlue™ (DAPI)	Ready-to-use	Thermo Fisher Scientific P36931
Goblet cells/ mucin	DyLight™ 649–conjugated Ulex europaeus agglutinin I (lectin)	1:200	Vector Laboratories DL-1068-1
Ki-67	Rabbit anti-Ki-67	1:250	Abcam ab15580
Secondary antibody	Alexa Fluor™ 647–conjugated goat anti-rabbit IgG	1:1000	Invitrogen A21244

Supplementary Movie 1.

Three-dimensional confocal Z-stacks reconstructed with Volocity software show sfGFP-expressing ETBF aggregates within distal colonic ACF lesions of 8-week-old *Apc*$^{Min/+}$ mice following neonatal ETBF colonization. DAPI (blue) and F-actin (white) outline crypt architecture, highlighting the spatial association of ETBF with early neoplastic crypts. Imaged at 20× magnification.

Supplementary Movie 2.

Same as Movie S1, imaged at 40× magnification.
